# Nutrient homeostasis within the plant circadian network

**DOI:** 10.3389/fpls.2015.00299

**Published:** 2015-04-29

**Authors:** Michael J. Haydon, Ángela Román, Waheed Arshad

**Affiliations:** Department of Biology, University of York, York, UK

**Keywords:** circadian clocks, *Arabidopsis*, plants, nutrient, micronutrient, ion, metal, carbohydrate

## Abstract

Circadian clocks have evolved to enhance adaptive physiology in the predictable, fluctuating environment caused by the rotation of the planet. Nutrient acquisition is central to plant growth performance and the nutrient demands of a plant change according to the time of day. Therefore, major aspects of nutrient homeostasis, including carbon assimilation and mineral uptake, are under circadian control. It is also emerging that there is feedback of nutritional status to the circadian clock to integrate these processes. This review will highlight recent insights into the role of the circadian clock in regulating plant nutrition as well as discuss the role for nutrients in affecting circadian function.

## Introduction

Nutrient homeostasis drives physiology, development and cellular metabolism (Clemens et al., 2002; Amtmann and Blatt, 2009). Nutrient elements are required to synthesize organic macromolecules and various nutrient ions fulfill structural or catalytic roles in key proteins or act as cofactors or signaling molecules. Plants are exposed to extreme daily fluctuations in the environment that dramatically affect physiology and metabolism. For example, there are rhythmic changes in nutrient demands to drive photosynthesis in chloroplasts and daily rhythms in transpiration rates alter the major nutrient transport pathways through the xylem. Therefore, nutrients need to be continuously mobilized between tissues and organelles, particularly in conditions of nutrient scarcity.

**FIGURE 1:**
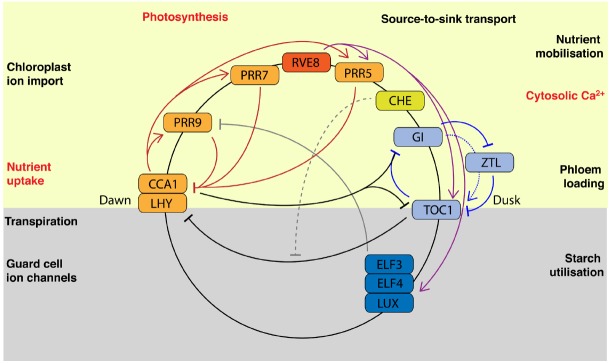
**Circadian regulated nutrient homeostasis.** The current model of the *Arabidopsis* circadian clock is comprised of multiple interlocking regulatory feedback loops and is described in detail elsewhere (Hsu and Harmer, 2014). Briefly, morning-expressed *CIRCADIAN CLOCK ASSOCIATED1 (CCA1)* and *LATE ELONGATED HYPOCOTYL (LHY)* and afternoon expressed *REVEILLE8 (RVE8)* encode related Myb-like transcription factors that activate transcriptional repressors in a time-of-day dependent manner. Thus, the transcriptional repressors *PSEUDO-RESPONSE REGULATOR9 (PRR9)*, PRR7, PRR5, and PRR1/TIMING OF CAB1 (TOC1) are expressed sequentially to act on *CCA1* and *LHY*. CCA1 HIKING EXPEDITION (CHE) inhibits repression of CCA1 by TOC1. The evening-expressed GIGANTEA regulates TOC1 by stabilizing the F-box protein ZEITLUPE (ZTL). EARLY FLOWERING3 (ELF3), ELF4, and LUX ARRYHTHMO (LUX) form the “evening complex,” which is activated by RVE8 and represses PRR9. The circadian regulated nutrient homeostasis processes that are discussed in this review are shown. The position represents approximate time of day of maximal activity and therefore illustrates the temporal relationship to components of the circadian oscillator. All these processes contribute to rhythmic physiology but some also affect circadian clock function directly or indirectly. Those processes for which direct regulation of circadian rhythms has been demonstrated are highlighted in red text.

The circadian clock regulates rhythmic growth and physiology (Hsu and Harmer, 2014). Circadian clocks are molecular oscillators comprised of interlocking regulatory feedback loops. The components of the clock are set, or “entrained,” by external cues such as light and temperature and form a time-keeping mechanism to predict daily and seasonal changes in the environment and directly regulate rhythmic physiological outputs. The current model for the oscillator in *Arabidopsis thaliana* (Figure 1) is characterized by a network of transcriptional regulators expressed at different times of day. The general architecture of circadian clocks is expected to be broadly conserved across higher plants (Song et al., 2010).

Transpiration generates the major pathway for nutrient movement through the xylem. This rhythmic process is driven by regulation of stomatal aperture and coordinated by activity of aquaporins, both of which are regulated by the circadian clock (Dodd et al., 2004, 2005; Takase et al., 2011; Caldeira et al., 2014). Photosynthesis, which is also circadian regulated (Harmer et al., 2000; Dodd et al., 2005; Noordally et al., 2013), creates significant demand for nutrients in chloroplasts, as well as driving nutrient movement through the phloem. There is extensive transcriptional regulation of nutrient transport processes in *Arabidopsis* (Haydon et al., 2011) but we currently know little about circadian-driven rhythmic fluxes of nutrients. Recent research has highlighted important roles for rhythmic metabolism in circadian entrainment, including photosynthetic outputs in plants (Haydon et al., 2013b). There is also evidence that nutrients other than carbon affect plant circadian clock function by directly altering the circadian oscillator (Dodd et al., 2007; Gutiérrez et al., 2008; Chen et al., 2013; Hong et al., 2013; Salomé et al., 2013), or indirectly through effects on rhythmic physiology (Lebaudy et al., 2008; Gilliham et al., 2011; Caldeira et al., 2014). In this mini-review, we highlight examples of regulation of nutrient homeostasis by the clock and summarize evidence of whether any of these nutrients contribute to the complex circadian network.

## Carbon

Photosynthesis provides the energy and the structural components to build cells. This defining aspect of metabolism in the green lineage generates huge nutrient demands (Shcolnick and Keren, 2006) and much of plant physiology converges on this process. Glucose is synthesized diurnally, converted to sucrose for transport and stored as starch in plastids. This stored starch is then converted back into soluble sugars during the night to support nocturnal metabolism and growth. In *Arabidopsis* leaves, the rate of starch degradation at night is linear, and almost all of the starch is used by dawn (Smith and Stitt, 2007). Photosynthesis and starch metabolism, including associated transport processes, are regulated by the circadian clock (Harmer et al., 2000; Dodd et al., 2005; Graf et al., 2010; Noordally et al., 2013), but the exact mechanism(s) of this regulation remains unknown.

Sugars can influence the circadian oscillator. Daily rhythms in carbohydrate metabolism contribute to oscillations in a wide range of transcripts in *Arabidopsis* and modify the expression of over half of the circadian-regulated transcriptome (Bläsing et al., 2005). Addition of exogenous sugars to growth medium shortens circadian period in *Arabidopsis* seedlings grown in continuous light (Knight et al., 2008; Haydon et al., 2013b) and can sustain circadian rhythms in continuous dark (Dalchau et al., 2011). Pulses of exogenous sucrose can advance or delay circadian phase, depending on the time of day (Haydon et al., 2013b). Opposite to the effect of exogenous sugars, inhibition of photosynthesis lengthens circadian period through de-repression of the clock gene *PSEUDO-RESPONSE REGULATOR7* (PRR7; Haydon et al., 2013b), which encodes a repressor of *CIRCADIAN CLOCK ASSOCIATED1 (CCA1*; Figure 1). PRR7 directly regulates genes involved in metabolism and abiotic stress (Liu et al., 2013). It was proposed that by acting on *PRR7*, rhythmic production of endogenous sugars from photosynthesis contributes to circadian entrainment, providing feedback to the clock to optimize rhythmic nutrient metabolism. Similarly, a role for mobile photosynthate has been proposed to drive circadian rhythms in roots of *Arabidopsis* in the absence of light and photosynthesis (James et al., 2008), suggesting a role for carbohydrate transport in circadian clock function.

## Macronutrients: Nitrogen, Phosphorous, and Sulfur

There is circadian regulation of transporters for nitrogen (N), phosphorous, and sulfur (Haydon et al., 2011). Transcripts for chloroplast importers for sulfate and phosphate (Versaw et al., 2002; Cao et al., 2013) peak around dawn (Dodd et al., 2007; Covington et al., 2008; Wang et al., 2011), consistent with increased demands for photosynthesis during the day. Transcripts encoding proteins for nitrate (Ho et al., 2009) and ammonium uptake (Gazzarrini et al., 1999) have peak expression around dawn whereas transcripts for phloem loading of nitrate peak after dusk to drive source-to-sink movement of N in the night (Fan et al., 2009).

A direct link between the circadian oscillator and N metabolism has been demonstrated (Gutiérrez et al., 2008). Network analysis of a transcriptome dataset of responses to assimilated organic N metabolites in *Arabidopsis* identified the clock component CCA1 as a putative “master regulator” in the N-regulated network. CCA1 was shown to bind to promoters of N-assimilation genes and alter their expression, demonstrating direct regulation of N metabolism by the clock. Furthermore, pulses of N caused stable phase shifts in *CCA1* expression, suggesting feedback of N status to the circadian clock (Gutiérrez et al., 2008). It is not known whether long-term changes in N status affect circadian rhythms in plants, but N deficiency shortened circadian period in the photosynthetic marine dinoflagellate, *Gonyaulax polyedra* (Sweeney and Folli, 1984). Mutants of *Glycine max* with impaired nodule development had altered expression of clock transcripts in nodules, which might be due to altered plant N status (Chiasson et al., 2014).

## Macronutrient Ions: Calcium, Magnesium, and Potassium

Calcium (Ca) is a highly abundant nutrient ion, comprising around 3% of leaf dry weight. Ca movement is dependent on the transpiration stream and is stored at high concentrations in vacuoles and the apoplasm, where a large proportion of this is contained within the pectin component of the cell wall (Gilliham et al., 2011). Ca^2+^ ions also act as second messengers in cell signaling, contributing to a wide array of physiological processes (Dodd et al., 2010). It has been proposed that Ca^2+^ might directly regulate water flow by acting on aquaporins, which would affect nutrient movement through the plant (Gilliham et al., 2011).

There are circadian oscillations in the concentration of cytosolic free Ca^2+^ ([Ca^2+^]_cyt_) in plants, peaking around dusk (Johnson et al., 1995; Love et al., 2004). These oscillations are specific to leaf mesophyll cells (Martí et al., 2013). Treatment of *Arabidopsis* with nicotinamide abolishes circadian rhythms of [Ca^2+^]_cyt_ and lengthens circadian period by 2–4 h (Dodd et al., 2007). This is consistent with a role for these Ca^2+^ signals in regulating the circadian network, although the target(s) of these are unknown. Nicotinamide has also been proposed to affect the plant circadian network by altering histone modifications on clock gene promoters (Malapeira et al., 2012). Both effects are possible, since nicotinamide affects multiple aspects of NAD^+^ metabolism (Haydon et al., 2013a). Transcripts for several Ca^2+^ channels and transporters are regulated by the clock, some of which peak around dusk (Haydon et al., 2011), but regulation of the transport mechanisms driving circadian oscillations of [Ca^2+^]_cyt_ are likely to occur post-transcriptionally. It has been proposed that circadian oscillations of [Ca^2+^]_cyt_ originate from cADPR-activated channels, most likely in the ER or vacuole (Dodd et al., 2007). There are no plant orthologs for ryanodine receptors, the cADPR-activated channels in animal cells, and so the identity of the elusive plant receptor(s) or channel(s) remains unknown (Dodd et al., 2010).

Potassium (K) is the most abundant cation in plants, fulfilling roles in enzyme activation, membrane transport, and osmoregulation (Wang and Wu, 2013). Thus, K impacts on key aspects of rhythmic physiology, most notably on water and solute fluxes. There is circadian regulation of transcripts for several K^+^ transporters in *Arabidopsis* (Haydon et al., 2011) but these do not represent the major, characterized acquisition pathways. *Arabidopsis* mutants lacking inward K^+^ channel activity in guard cells were impaired in their ability to increase transpiration rates in anticipation of dawn and had reduced biomass when grown in high light intensity (Lebaudy et al., 2008). These data highlight the potential impact of specific circadian regulated ion fluxes on rhythmic physiology and plant growth.

Magnesium (Mg) fulfills two major roles in plants: at the center of the chlorin ring of chlorophyll and in the active form of ATP. Both of these roles are critical for photosynthesis and create a high demand for Mg in chloroplasts, particularly during the light period. However, as energy demands for cellular metabolism change throughout the day, mobilization of Mg may be required. Any role for the circadian clock in regulation of Mg transport remains uncovered.

A primary symptom of Mg deficiency is a defect in sugar partitioning, rather than primary production (Hermans et al., 2006), perhaps reflecting the relative importance of ATP-driven transport processes over chlorophyll concentration. Transcriptome profiling of Mg-deficient *Arabidopsis* plants identified changes in expression of clock gene transcripts (Hermans et al., 2010a,b). Phase of *CCA1* and *LATE ELONGATED HYPOCOTYL (LHY)* expression was delayed in Mg deficient plants grown in light/dark cycles. This might equate to a lengthened circadian period in continuous light conditions and might be due to impaired sugar production or partitioning, consistent with the role of sugars in circadian entrainment (James et al., 2008; Haydon et al., 2013b). Alternatively, the effects of Mg deficiency on circadian clock gene expression could be due to other energy-dependent processes.

## Micronutrients: Copper and Iron

Micronutrients such as iron (Fe), zinc, manganese and copper (Cu) fulfill structural and/or catalytic roles in a wide range of proteins. They play roles in chloroplast function and photosynthesis and the requirements for metal ions are orders of magnitude higher in photosynthetic organisms (Shcolnick and Keren, 2006). These micronutrients are also highly cytotoxic, and so the concentrations of unbound ions must be tightly controlled (Clemens et al., 2002). They are often scarcely available in soils and are required to be delivered to various cellular compartments throughout the plant, which depends on transpiration and energy-dependent transport. When availability is low, elements must be mobilized to match the changing needs of metabolism across the daily environmental cycle (Blaby-Haas and Merchant, 2013). It is therefore likely that there are daily rhythms in the concentration and distribution of micronutrients in different tissues and organelles.

There is circadian regulation of transcripts for Cu transporters and their promoters contain conserved elements for circadian clock regulation (Perea-García et al., 2010). Plasma membrane transporters tend to peak in expression around subjective dawn, whereas chloroplast-localized transporters peak later in the day (Dodd et al., 2007; Covington et al., 2008). Cu toxicity reduced amplitude, and perhaps lengthened circadian period, of circadian clock gene expression and overexpression of Cu uptake transporters *COPPER TRANSPORTER 1 (COPT1)* or *COPT3* enhanced sensitivity to Cu toxicity in continuous light compared to light/dark cycles (Andrés-Colás et al., 2010). These Cu-dependent phenotypes in continuous light might relate to recent findings that Cu homeostasis interacts directly with light signaling pathways (Zhang et al., 2014). Although it is clear that there is rhythmic regulation of plant Cu homeostasis by the circadian clock, it remains uncertain whether Cu has a direct impact on the circadian network.

Similar to Cu transporters, transcripts for Fe transport and storage have circadian rhythms in expression (Duc et al., 2009; Hong et al., 2013). However, the extent of circadian-regulated Fe homeostasis is probably under-estimated since transcripts for many Fe transporters are only expressed in Fe deficient plants (Vert et al., 2002) and might not be represented in published circadian time-course datasets (Dodd et al., 2007; Covington et al., 2008). There is circadian regulation of transcripts for FERRITIN (FER), which are regulated by Fe and are involved in cellular responses to excess Fe and oxidative stress (Ravet et al., 2009). *FER1*, *FER3*, and *FER4* are directly regulated by PRR7 and overexpression of *PRR7* increases sensitivity to excess Fe (Liu et al., 2013). Forward genetic screens for regulators of *FER1* (Duc et al., 2009) identified a mutant in *TIME FOR COFFEE (TIC)*, a component of the circadian network (Ding et al., 2007). *tic* mutants are strongly impaired in a range of metabolic phenotypes, including redox homeostasis (Sanchez-Villarreal et al., 2013), and so the finding that *tic* mutants alter expression of *FER1* might be related to roles for TIC in oxidative stress.

Several recent studies have revealed an effect of plant Fe status on the plant circadian network (Chen et al., 2013; Hong et al., 2013; Salomé et al., 2013). Circadian period is lengthened by *ca.* 1–2 h in Fe-deficient *Arabidopsis* seedlings (Chen et al., 2013; Hong et al., 2013; Salomé et al., 2013). This effect is light-dependent, and requires protein translation in plastids, pointing to an involvement of chloroplasts in circadian responses to Fe status (Chen et al., 2013; Salomé et al., 2013). The chloroplasts are the primary site for Fe in *Arabidopsis* leaves (Shikanai et al., 2003) and a major symptom of Fe deficiency is leaf chlorosis, which is associated with impaired chloroplast function (Varotto et al., 2002). Since direct inhibition of photosynthesis lengthens circadian period by 2–4 h (Haydon et al., 2013b), the effect of Fe deficiency might also be through inhibition of photosynthetic sugar production acting on PRR7. However, this seems unlikely since at least two of the studies on circadian Fe responses included sugar in the growth media (Hong et al., 2013; Salomé et al., 2013), which suppresses the effect of inhibition of photosynthesis on circadian period (Haydon et al., 2013b). Similarly, unlike the effect of sugar, Fe dependent effects on circadian period are unaffected in *prr7* or *cca1* mutants (Chen et al., 2013; Haydon et al., 2013b; Salomé et al., 2013). Therefore, Fe status appears to regulate the clock by a distinct mechanism from that of photosynthetically-derived sugars acting on *PRR7* (Haydon et al., 2013b). Mutants in *GIGANTEA* (*GI*) or *ZEITLUPE* (*ZTL*) were reported to be insensitive to Fe dependent circadian effects (Chen et al., 2013; Hong et al., 2013). The clock proteins ZTL and GI physically interact (Kim et al., 2007), and a role for GI in sugar dependent circadian rhythms has been proposed (Dalchau et al., 2011). Circadian Fe responses might act through these evening-expressed clock components.

A role for Fe in the circadian network in animals has been described. The Fe-containing cofactor, haem, binds to REV-ERBα, a core component of the mammalian circadian clock (Yin et al., 2007). There are circadian rhythms in haem levels in animals (Kaasik and Lee, 2004) and plants (Salomé et al., 2013), but no target for haem-binding in the plant circadian clock has been identified. Nevertheless, free haem levels are modestly elevated in low Fe conditions in *Arabidopsis* and haem biosynthesis mutants do have altered circadian period (Salomé et al., 2013), although it is difficult to predict the effects on cellular haem levels in these mutants. Whether there is a direct role for haem in the plant circadian network remains an open question.

Haem is an intermediate in the tetrapyrrole biosynthesis pathway. There is circadian regulation of this branched pathway (Covington et al., 2008; Salomé et al., 2013), which produces chlorophyll and phytochromobilin, as well as contributing to plastid-to-nucleus retrograde signaling via intermediates (Koussevitzky et al., 2007; Woodson et al., 2011). Thus, the roles of this pathway in cellular metabolism are varied and complex. The effects of norflurazon and lincomycin, chemical agonists of retrograde signaling, on circadian period are inconsistent across studies, perhaps due to differing growth conditions (Chen et al., 2013; Haydon et al., 2013b; Salomé et al., 2013). Similarly, mutants in some but not all components of tetrapyrrole-dependent retrograde signaling pathways were impaired in the circadian Fe response (Salomé et al., 2013). The loss of circadian Fe responses in *phytochromeA (phyA) phyB* mutants might implicate phytochromobilin (Salomé et al., 2013). Altogether, these recent studies on circadian Fe responses are intriguing but further experiments are required to define a role for Fe status in circadian time-keeping. It remains uncertain whether these observations describe a specific effect of Fe, or a more general, indirect effect of metabolic stress in chloroplasts.

## Conclusion

There is accumulating evidence of circadian regulation of many aspects of plant nutrient homeostasis that have ranging effects on physiology and metabolism. There is circadian regulation of transcripts associated with nutrient acquisition and transport, and several examples of feedback regulation of the circadian clock by nutrients, as well as indirect effects of nutrients on rhythmic physiology. Together, these illustrate a complex network of interactions between the circadian oscillator, nutrient status and rhythmic physiology. However, with a few notable exceptions, surprisingly little has been reported for circadian rhythms in nutrient concentrations in plants. This might be because there is little change in whole tissues, making these difficult to measure with current quantitative techniques. As more nutrient biosensors and imaging tools become available in plants (Grossmann et al., 2011; Krebs et al., 2012; Martí et al., 2013; Lanquar et al., 2014) it might be possible to investigate daily nutrient fluxes with higher spatial and temporal resolution in specific tissue types or subcellular compartments (Haydon, 2014). The importance of tissue-specificity of oscillators in plant circadian clock function is emerging (Wenden et al., 2012; Martí et al., 2013; Endo et al., 2014) and it seems feasible that dynamic nutrient fluxes, which are tightly linked to rhythmic physiology, could play an important role.

## Author Contributions

MH, ARF, and WA all contributed to the content, drafting and editing of the manuscript.

### Conflict of Interest Statement

The authors declare that the research was conducted in the absence of any commercial or financial relationships that could be construed as a potential conflict of interest.
